# Postnatal Development of the Subcellular Structures and Purinergic Signaling of Deiters’ Cells along the Tonotopic Axis of the Cochlea

**DOI:** 10.3390/cells8101266

**Published:** 2019-10-17

**Authors:** Eszter Berekméri, Ádám Fekete, László Köles, Tibor Zelles

**Affiliations:** 1 Department of Pharmacology and Pharmacotherapy, Semmelweis University, Nagyvárad tér 4., 1089 Budapest, Hungary; berekmeri.eszter@med.semmelweis-univ.hu (E.B.); koles.laszlo@med.semmelweis-univ.hu (L.K.); 2 Department of Ecology, University of Veterinary Medicine, Rottenbiller u. 50., 1077 Budapest, Hungary; 3 Program in Neurosciences and Mental Health, The Hospital for Sick Children, 555 University Ave, Toronto, ON M5G 1X8, Canada; fekadam001@gmail.com; 4 Department of Pharmacology, Institute of Experimental Medicine, Hungarian Academy of Sciences, Szigony u. 43., 1083 Budapest, Hungary

**Keywords:** mouse hemicochlea, single-cell electroporation, subcellular Ca^2+^ imaging, Deiters’ cells, postnatal development, tonotopy, ATP

## Abstract

Exploring the development of the hearing organ helps in the understanding of hearing and hearing impairments and it promotes the development of the regenerative approaches-based therapeutic efforts. The role of supporting cells in the development of the organ of Corti is much less elucidated than that of the cochlear sensory receptor cells. The use of our recently published method of single-cell electroporation loading of a fluorescent Ca^2+^ probe in the mouse hemicochlea preparation provided an appropriate means to investigate the Deiters’ cells at the subcellular level in two different cochlear turns (apical, middle). Deiters’ cell’s soma and process elongated, and the process became slimmer by maturation without tonotopic preference. The tonotopically heterogeneous spontaneous Ca^2+^ activity less frequently occurred by maturation and implied subcellular difference. The exogenous ATP- and UTP-evoked Ca^2+^ responses were maturation-dependent and showed P2Y receptor dominance in the apical turn. By monitoring the basic structural dimensions of this supporting cell type as well as its spontaneous and evoked purinergic Ca^2+^ signaling in the hemicochlea preparation in different stages in the critical postnatal P5-25 developmental period for the first time, we showed that the soma and the phalangeal process of the Deiters’ cells go through age- and tonotopy-dependent changes in the morphometric parameters and purinergic signaling.

## 1. Introduction

Exploring the development of the hearing organ helps to better understand the (patho)physiology of hearing and might contribute to develop novel regenerative therapeutic approaches [1,2] for restoring impaired hearing function [3].

Mouse, the primary animal model in hearing research, similarly to other altricial species, are deaf at birth [4,5]. Up to postnatal day 5–7 (P5–7), among others, important maturation steps are finalized in the inner (IHC) and outer hair cells (OHC) and their innervation [6,7,8,9]. The first behavioural and auditory nerve responses that coincide with the opening of the outer ear can be observed at P10. In the next 4–5 days, the hearing rapidly matures. Up to P14–15, the amplitudes of the cochlear potentials and the eighth nerve action potentials increase, the optimum of sensitivity at 15 kHz appears, and the frequency range on the audiogram extends. Although many studies consider this age as the onset of mature hearing, the last mitosis events occur in the organ of Corti by P18 [4,10,11,12]. During the P20–25 period, the cochlea develops further both in morphology [11] and functions [4]. In this study we investigated the postnatal development of the cochlea, encompassing the period from the early prehearing (P5–7) to the late posthearing onset stage (P20–25) and aimed to remedy the shortcomings of most studies on the development of hearing that are restrained to the embrionic and neonatal or prehearing developmental periods (<P10) [1,7,8,13]. Furthermore, these studies primarily focus on the receptor cells, the auditory nerves, and their synaptic connections [6,8,14,15,16], but the supporting cells of the cochlea receive much less attention.

Mechanoelectrical transduction channels in hair cells are inducible from P0 in the basal and from P2 in the apical turn of the cochlea [17]. Ion channels that are necessary for electrical activity and firing of spontaneous action potentials appear by the first postnatal week in a strict order along the cochlear duct [6,18,19]. The development and tonotopic gradient of morphological and functional properties of supporting cells in the organ of Corti are also necessary for the mammalian cochlea to permit amplification and frequency discrimination in hearing at a wide range of sound frequencies. However, their development is less well characterized than that of the hair cells. Their structural and functional development in the context of purinergic signaling has not been investigated yet, contrary to their indispensable role in the cochlear structure and function.

ATP is an important extracellular purinergic mediator [20,21,22] in the hearing organ [23,24]. During the prehearing phase of cochlear development, ATP is released by immature supporting cells through hemichannels and it initiates spontaneous Ca^2+^ action potential firing in IHCs [7,23,25,26]. This sensory input-independent firing of IHCs is fundamental for the maturation, pruning, and maintenance of the hair cell—auditory neuron synapses [7,8,25,27]. A recent study demonstrated that this autocrine and paracrine purinergic signaling does not only drive the development of IHCs in the greater epithelial ridge (GER, Kölliker’s organ), but it is also crucial for OHC maturation in the lesser epithelial ridge (LER) [28]. Deiters’ cells, the supporting cells in the LER, are paramount players in this process.

Deiters’ cells have both ionotropic (P2X) and metabotropic (P2Y) purinergic receptors and their activation induces intracellular Ca^2+^ responses [29,30,31]. Deiters’ cells provide physical scaffold, metabolic support in the organ of Corti, modulate OHC electromotility [32,33], and actively contribute to cochlear amplification [32,34,35]. Movements of their phalangeal processes can be evoked by ATP, and they may influence the active cochlear micromechanics [30,31,33,36]. As the subcellular compartments of Deiters’ cells (phalangeal process and soma) have never been studied in a developmental context, we performed Ca^2+^ imaging on the phalangeal processes and somata between P5 and 25 and examined the spatial and tonotopic organization of purinergic signaling.

In this paper, we monitored the postnatal development-dependent changes in morphometric parameters, spontaneous Ca^2+^ spiking, and ATP-evoked Ca^2+^ transients in Deiters’ cells. The hemicochlea preparation [37,38] and the use of Ca^2+^ indicator electroporation loading of single Deiters’ cells in it, as introduced in our laboratory [30], allowed us, for the first time, to perform these measurements in different subcellular compartments (soma, phalangeal process), in two different cochlear turns (apical, middle) and in the critical P5-25 developmental period of the cochlea.

## 2. Materials and Methods

### 2.1. Tissue Preparation

All animal care and experimental procedures were in accordance with the National Institute of Health Guide for the Care and Use of Laboratory Animals. The Animal Use Committee of Semmelweis University, Budapest, approved procedures. Hemicochlea preparation was made from acutely dissected cochleae of BALB/c mice from P5 to P25. Preparation was carried out as it has been described by the Dallos’ group [37] and was adopted in our laboratory [30,31]. Briefly, after decapitation in isoflurane anaesthesia, the head was divided in the midsagittal plane, and the cochleae were removed and placed in ice-cold solution similar to perilymph of adult mice (composition in mM: NaCl 22.5; KCl 3.5; CaCl_2_ 1; MgCl_2_ 1; Hepes 10; Na-gluconate 120; glucose 5.55; pH 7.4; 320 mOsm/L). The solution was continuously oxygenated. Medial surface of each cochlea was glued (Loctite 404, Hartford, CT) onto a plastic plate with the diameter of 7 mm and placed in a cutting chamber of a vibratome (Vibratome Series 3000, Technical Products International Inc., St. Louis, Mo, USA) while being continuously bathed in the ice-cold solution. Cochlea was cut half in the modiolar plane (Feather Microtome Blade R35, CellPath Ltd., Newtown, UK) under visual control through a stereomicroscope (Olympus SZ2-ST, Olympus Corporation, Philippines) and only the glued half was used for the experiments.

### 2.2. Targeted Single-Cell Electroporation

Single-cell electroporation was carried out as it was previously set up by our group in hemicochlea preparation [30]. Briefly, hemicochlea was placed into an imaging chamber that was filled and perfused (speed: 3.5 mL/min) with the oxygenated experimental solution on the microscope-stage. Deiters’ cells in the organ of Corti were chosen under a LUMPlanFl 40×/0.80w water immersion objective (Olympus, Japan) with oblique illumination. Borosilicate pipettes (5–7 MΩ; Harvard Apparatus, Holliston, MA, United States) were filled with the Ca^2+^ indicator Oregon Green 488 BAPTA-1 hexapotassium salt (OGB-1) (ThermoFisher Scientific) dissolved in distilled water at a final concentration of 1 mM. The pipettes were mounted onto an electrode holder that was attached to a micromanipulator (Burleigh PCS-5000, Thorlabs, Munich, Germany). The pipette under visual control approached each chosen cell; a single square wave current impulse (10 ms, 10 µA) load the cells with the Ca^2+^ indicator. The pulses were generated by pCLAMP10 software-guided stimulator system (Biostim STE-7c, Supertech Ltd., Pecs, Hungary; MultiClamp 700B Amplifier and Digidata 1322A, Molecular Devices, Budapest, Hungary). We excluded the data that were recorded from the basal turn Deiters’ cells because the retention of the electroporated dye was low.

### 2.3. Calcium Imaging

The experiments were carried out at room temperature. The cells were illuminated by 494 ± 5 nm excitation light (Polychrome II monochromator, TILL Photonics, Germany) and the emitted light was monitored after passage through a band-pass filter (535 ± 25 nm). Fluorescent images were obtained with an Olympus BX50WI fluorescence microscope (Olympus, Japan) equipped with a Photometrics Quantix cooled CCD camera (Photometrics, USA). The system was controlled with the Imaging Workbench 6.0 software (INDEC BioSystems, USA). The image frame rate was 1 Hz during the drug-evoked responses and 0.1 Hz otherwise to reduce phototoxicity and photobleaching. Regions of interests with a similar area were drawn in the soma of the stained cell and the phalangeal process of Deiters’ cell imaging. The whole experiment was performed within 1.5–2 h after decapitation. Cells with not preserved morphology were excluded from further analysis.

### 2.4. Measurement of the Morphological Parameters

**Figure 1:**
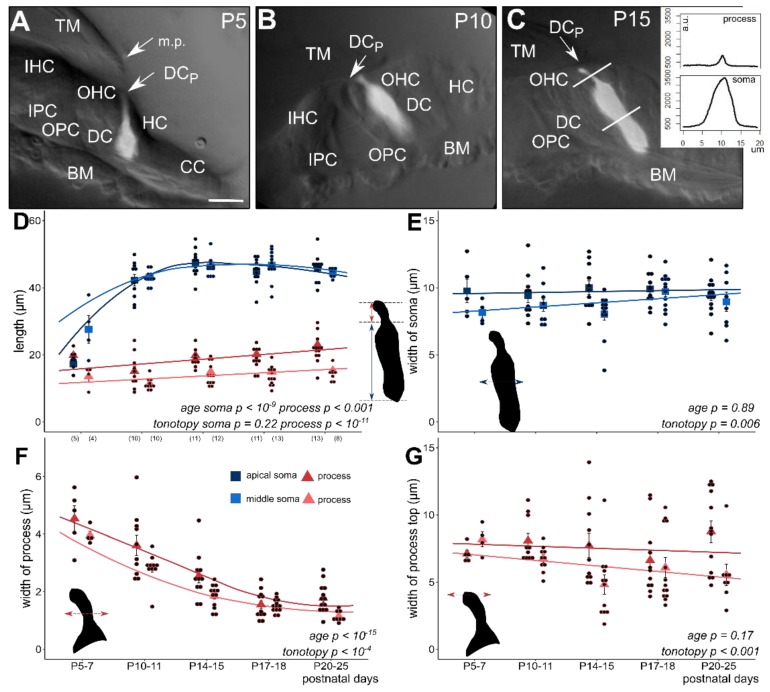
Morphometric changes of the soma and the phalangeal process of apical and middle turn Deiters’ cells during postnatal development (P5–25). The subcellular compartments (soma and phalangeal process) were visualized by single-cell electroporation loading of a fluorescent Ca^2+^ indicator (OGB-1) in the BALB/c mouse hemicochlea. Image of fluorescent cells was overlaid on the respective widefield image illuminated obliquely (**A**–**C**, all in middle turn). Plot of fluorescence intensities along a line profile was used to measure the appropriate cell dimensions (inset in **C**). In P5–7 mice the tunnel of Corti is not opened yet and the marginal pillars can be seen over the third row of Deiters’ cell (**A**, P5). The cell shape differs totally from the one of the older Deiters’ cells (**B**, P10 and **C**, P15). From P10 the tunnel of Corti is opened (**B**,**C**). At this developmental stage the soma elongated significantly, nearly to the adult level (**D**); apical, dark blue square; middle, light blue square). The maturation-dependent elongation of the phalangeal process was gradual and showed tonotopic difference between the apical and middle cochlear turn (**D**); apical, dark red triangle; middle, light red triangle). Contrary to the elongation in length, the width of the phalangeal processes decreased gradually by maturation during the P5–25 period and also showed a tonotopic difference, apical turn processes being wider (**F**). Width of the soma (**E**) and of the head plate of the phalangeal process (**G**) did not change significantly during the development, but the turn differences were present in both parameters, i.e., cells in the apical turn showed the wider soma and head plate in all ages compared to middle turn Deiters’ cells. The width of the subcellular compartments was measured at their half-length. Square and triangle symbols show mean ± SEM and the black dots indicate the individual measurements. Number of experiments are in parenthesis (**D**) and they are the same for the appropriate variables in E–G. Respective *p* values, showing the significance of age or tonotopy are written in italics. Details of statistical analysis are given in the Materials and Methods. The scale bar represents 15 µm. TM: tectorial membrane; BM: basal membrane; IHC: inner hair cell; OHC: outer hair cell; IPC: inner pillar cell; OPC: outer pillar cell; DC: soma of Deiters’ cell; DCp: process of Deiters’ cell; HC: Hensen’s cell; CC: Claudius’ cell; m.p.: marginal pillar.

Right after electroporation z-stack of both fluorescent and widefield images (7–9 each) of all investigated Deiters’ cells were taken from the lower to the upper edge of the cells. The fluorescent images of all loaded cells were taken at the highest excitation intensity of the system. After acquisition of a z-stack the XY projection was formed in FIJI [39] and the morphometric parameters that were used in the statistical analysis were measured on the fluorescent XY projection. This is because the phalangeal processes can only be recognized on the fluorescent images. Precise recognition of the edges of cells on fluorscent images is less reliable than on the bright field ones; therefore, the plot of fluorescence intensities along a line profile was used to help in measuring the appropriate cell dimension (inset in Figure 1C). In addition, soma parameters measured on the widefield image were always compared to parameters that were determined on fluorescent image of the same cell and, in case of difference higher than 5%, the cell was excluded from the morphometric analysis. The length of the soma was measured from the Deiters’ cell base-basal membrane border to the basal part of the outer hair cells and the length of the phalangeal processes was calculated from OHC base to the headplate of the process. The width of the soma and the process were determined at the half length of these structures.

### 2.5. Drug Delivery

ATP and UTP (Sigma-Aldrich, St. Louis, MO., USA) in 100 µM concentration were added to the perfusion for 30 s. The buffer volume in the perfusion chamber was about 1.9 mL. Before the first drug application, an at least 3-min. long baseline period was registered in each experiment. At least 10 min. had to be elapsed between two drug stimuli.

### 2.6. Data Analysis

Data analysis was performed off-line. Cell image intensities were background-corrected while using a nearby area devoid of loaded cells. Using OGB-1, the relative fluorescent changes were calculated, as follows:

(1) $$\frac{dF}{F_{0}}=\frac{F_{t}-F_{0}}{F_{0}}$$

where F_0_ is the fluorescent intensity of the baseline and F_t_ is the fluorescent intensity at time t.

The response amplitudes were defined as the maximal change in intensity. Response duration was defined as the time between half amplitude on ascent and descent of the transient. Area under the curves (AUCs) and average responses were calculated in Igor Pro 6.37.

The data are presented as mean ± standard error of the mean (SEM). The number of experiments (n) indicates the number of cells. Testing of significance was performed based on the multiple linear regression models that were carried out in R version 3.2.3., where one of the variables was the location of the cell (apical or middle turn; tonotopy) and the other explanatory variable was the postnatal days (age). The appropriate *p* values are given in the Results and in the figures written in italics. According to these types of statistics the *p* values explain that how much each variable contributes to the measured value of the dependent variables (e.g., measured amplitude, AUC, or spontaneous activity). These method does not compare dependent variables to each other. The period of P5–7 was not included in the models (except in case of the morphological development), as we hypostatised that the P10–11 period is a starting point in case of hearing functions. Figures were made in Igor Pro, R ggplot2 package and Inkscape.

## 3. Results

### 3.1. The morphological Changes in Deiters’ Cell Somata and Processes during Postnatal Development Have No Tonotopic Preference

As we described before, we visualized the Deiters’ cells by single-cell electroporation of Ca^2+^ indicators in the hemicochlea preparation [30] and investigated the morphometric changes in the soma and phalangeal process of Deiters’ cells from the first to the third postnatal week (P5–25), covering the entire period of mouse postnatal auditory maturation. The anatomical structure of the organ of Corti at P5–7 in BALB/c mice (Figure 1A) significantly differed from the one at P10–11 and older mice (Figure 1B, C). The hearing organ in P5–7 mice was more compact and the tunnel of Corti was still closed in both the middle and the apical turns. In some cases, marginal pillars could be recognized above the outer row of Deiters’ cells (Figure 1A).

At P5–7, the length of the Deiters’ cells soma and phalangeal process was similar in the apical turn of the cochlea, while the soma was already twice as long as the phalangeal process in the middle turn. The significant elongation of the somas was evident at P10–11 in both turns when their length nearly reached the mature stage (Figure 1D, Table 1). By the age of P14–15, the length of the somas was 47.44 ± 1.23 µm in the apical and 46.23 ± 0.76 µm in the middle turn and no further elongation could be detected by the end of the investigated period. We found that the elongation of the somata was significant (*p* < 10^−9^), and thus maturation-dependent but without any tonotopic difference between the apical and middle turns (*p* = 0.226). On the other hand, the width of the somas did not change with age (*p* = 0.89) but the tonotopic difference persisted during the whole P5–25 maturation period with thinner Deiters’ cells in the middle turn (*p* = 0.006; Figure 1E, Table 1).

The phalangeal processes were longer in the apical than the middle turn, being elongated moderately during development (*p* < 0.001), and maintained their tonotopic difference in this parameter (*p* < 10^−11^; Figure 1D, Table 1). The change in the width of the phalangeal process, measured at its half-length, was more characteristic during the postnatal maturation. The processes gradually became slimmer by age in both turn of the cochlea (*p* < 10^−15^; Figure 1F, Table 1). The slope of the decrease was similar in the two turns, but the processes in the apical turn were persistently and significantly wider (*p* < 10^−4^; Figure 1F, Table 1). The width of the headplate of the process, which contributes to the formation of the reticular lamina, did not change by developmental age (*p* = 0.17), but it was wider in the apical turn (*p* < 0.001; Figure 1G). It means that the width of the process at half-length and at the headplate were larger in the apical cochlear turn, but their development was not tuned by the tonotopy.

### 3.2. The Maturation-Dependent Spontaneous Ca^2+^ Activity Is Tonotopically Heterogeneous and Implies Subcellular Difference

**Figure 2:**
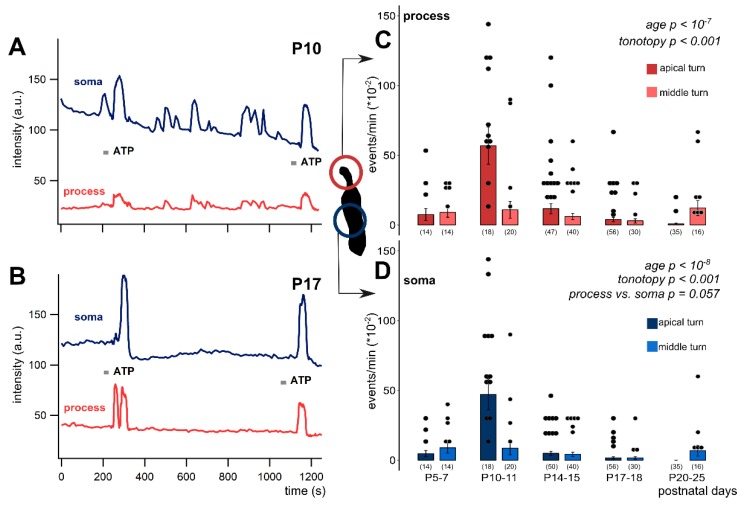
Age-dependent spontaneous Ca^2+^ activity in the apical and middle cochlear turn Deiters’ cells in the mouse hemicochlea preparation. Representative traces from apical turn cells demonstrate the remarkable difference in activity of the intracellular Ca^2+^ events at P10 (**A**) vs. P17 (**B**). Short grey lines indicate ATP application in the perfusion (100 µM, 30 s). Event activity of individual cells and averages of the activities by age are shown in the apical and middle turns in the phalangeal process (**C**) and the soma (**D**) of the Deiters’ cells. Spontaneous activity decreased significantly by age from P10-11 in both subcellular compartments. There was a prominent increase at P10-11 in the apical turn. The tonotopic difference was significant in both the process and the soma. The difference between the subcellular compartments was tendentious, i.e., the processes had higher event rate at all ages. Bars show mean ± SEM and the black dots indicate individual measurements. Values of zero (no spontaneous activity) were removed for clarity (high number of dots). Number of experiments are in parenthesis. Respective *p* values, showing the significance of age or turn differences or the process vs. soma difference are written in italics. Details of statistical analysis are given in the Materials and Methods.

Deiters’ cells showed spontaneous intracellular Ca^2+^ activity in their soma and phalangeal process. Spontaneous Ca^2+^ spikes appeared in both cochlear turns, but with different characteristics (Figure 2, Table 2).

In the apical turn, the frequency of the spontaneous events showed a prominent increase at P10-11, which gradually declined and virtually ceased at ≥P17 (Figure 2). The robust elevation in spontaneous Ca^2+^ transients observed in apical Deiters’ cells was not detected in the middle turn. In this turn, starting from a bit higher level at P5–7 (Table 2), the frequency of the Ca^2+^ events showed a continuous decrease with maturation (process: *p* < 10^−7^; soma: *p* < 10^−8^; Figure 2C,D) with a moderate increase at P20-25 (Table 2). The tonotopic difference was significant in both the process (*p* < 0.001) and the soma (*p* < 0.001). It is interesting to note that the frequency of Ca^2+^ events in the phalangeal processes tended to be higher (*p* = 0.057) than in the soma during the whole P5–25 maturation period (Table 2). These data imply that the spontaneous Ca^2+^ activity has subcellular dependency and it plays an important role in the tonotopic maturation of the cochlea around the hearing onset.

### 3.3. Exogenous ATP-Evoked Ca^2+^ Responses Are Maturation-Dependent

**Figure 3:**
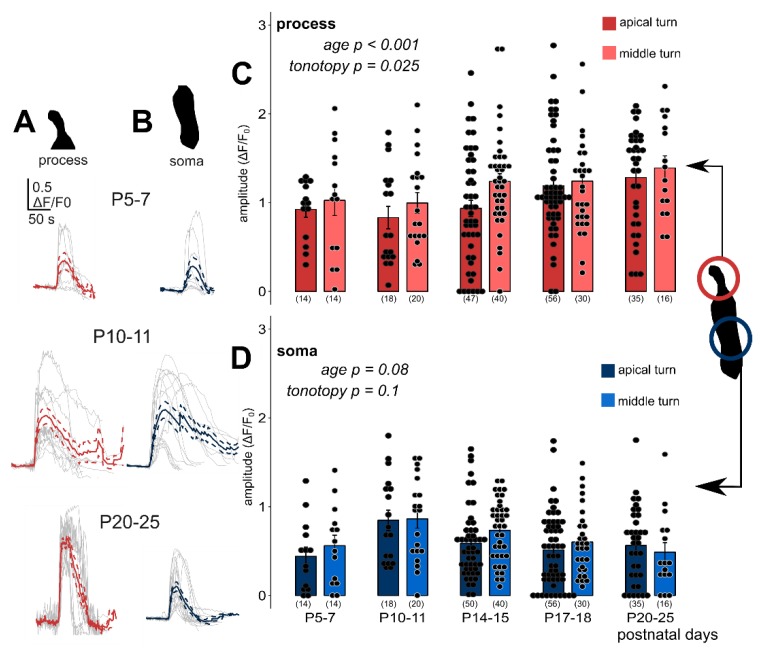
Exogenous ATP (100 µM in perfusion) evoked developmental state- and tonotopy-dependent Ca^2+^ transients in Deiters’ cells. Individual responses (grey) recorded in the phalangeal processes (**A**, red) and the somas (**B**, blue) of Deiters’ cells with their average (wider line) and ± SEM (dashed line) traces in the apical turn at three different developmental states. The bar graphs show the magnitudes of the amplitudes of all measured cell responses (black dots) and their mean ± SEM in the processes (**C**) and somas (**D**) in the apical (darker symbols) and middle (lighter symbols) cochlear turns. Note that the maturation- and turn difference-dependent gradual increase in the processes and the tendency of decrease in the somas from P10–11, which is the onset of mouse hearing. A number of experiments are in parenthesis. Respective *p* values, showing the significance of age or tonotopy are written in italics. Details of statistical analysis are given in the Materials and Methods.

Deiters’ cells were responsive for bath applied ATP (100 µM for 30 s in the perfusion) during the whole investigated maturation period (P5–25) in both apical and middle cochlear turns (Figure 3). The amplitude (dF/F_0_) of the phalangeal process Ca^2+^ responses showed a gradual increase by age from P10–11 (*p* < 0.001), and they were higher in the middle turn Deiters’ cells (*p* = 0.025; Figure 3A,C, Table 3). Responses in the soma seemed to follow an inverse correlation with age as far as amplitudes at P10–11, the onset of hearing were the highest and tended to decline by advancing age (*p* = 0.08; Figure 3B,D, Table 4). The tonotopy did not have a significant effect on amplitudes (*p* = 0.1), although the ATP responses in the middle turn somas were larger at all developmental states, except at P20–25 (Table 4). Response amplitudes in the prehearing P5–7 period did not fit to the age-dependent gradual rise in the process and decline in the soma, as they were a bit larger than the P10–11 values in the processes and lower than that in the somas (Figure 3C,D).

**Figure 4:**
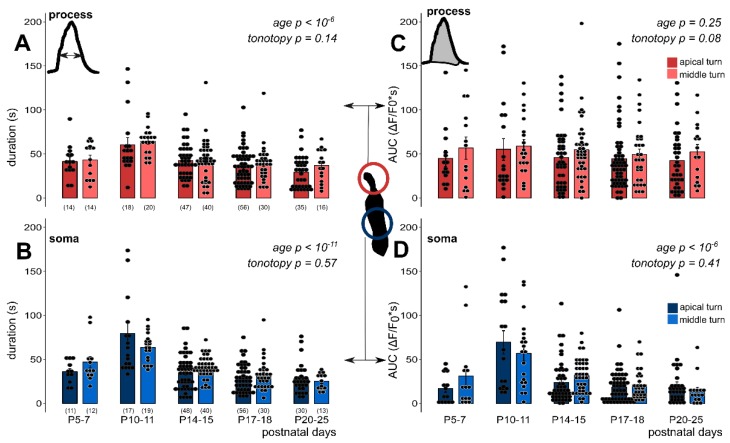
Duration and area under the curve (AUC) of exogenous ATP-evoked (100 µM) Ca^2+^ transients depend on the developmental state but lack tonotopic difference in phalangeal processes and somas of Deiters’ cells in the mouse hemicochlea preparation. The bar graphs show the durations (**A**,**B**) and AUC values (**C**,**D**) of all measured cell responses and their averages ± SEM in the processes (**A**,**C**) and somas (**B**,**D**) in the apical (darker colors) and middle (lighter colors) cochlear turns. Note the maturation-dependent gradual decrease in both the response duration and the AUC in both subcellular compartments (except in AUC in the process) from P10–11, which is the onset of mouse hearing. There was no significant turn difference between the apical and middle cochlear turn in either parameter or subcellular compartment. Number of experiments are in parenthesis (**A**,**B**) and they are the same for the appropriate variables in C and D. Respective *p* values, showing the significance of age or tonotopy are written in italics. Details of statistical analysis are given in the Materials and Methods.

We also analysed the maturation state and tonotopy dependence of the duration of responses and their AUCs (Figure 4, Table 3 and Table 4), which are characteristics of the response shape. Both parameters showed a developmental state-dependent decrease from the hearing onset (P10–11) in both subcellular compartments of the Deiters’ cells (response duration: *p* < 10^−6^ in the process, Figure 4A; *p* < 10^−11^ in the soma, Figure 4B; AUC: *p* < 10^−6^ in the soma, Figure 4D), except AUC in the phalangeal process, where only the tendency was recognizable (*p* = 0.25, Figure 4C). On the other hand, we did not measure significant tonotopic differences in either of these parameters in any of the subcellular compartments (response duration: *p* = 0.14 in the process, *p* = 0.57 in the soma; AUC: *p* = 0.08 in the process, *p* = 0.41 in the soma, Figure 4A–D).

However, consistently higher response duration and AUCs in all ages in the middle turn (vs. apical) Deiters’ cell processes can be recognized (Figure 4A,C, Table 3). Faster kinetics of the ATP-evoked Ca^2+^ transients after hearing onset indicates an increase in the precision of purinergic signaling, which might be important for a fast adaptation to sensory input modulation and the protection of the cochlea by limiting the Ca^2+^ load of the cells.

### 3.4. Selective P2Y Receptor Activation Evokes Maturation and Tonotopy-Dependent Ca^2+^ Transients with a P2Y Receptor Dominance in the Apical Turn

**Figure 5:**
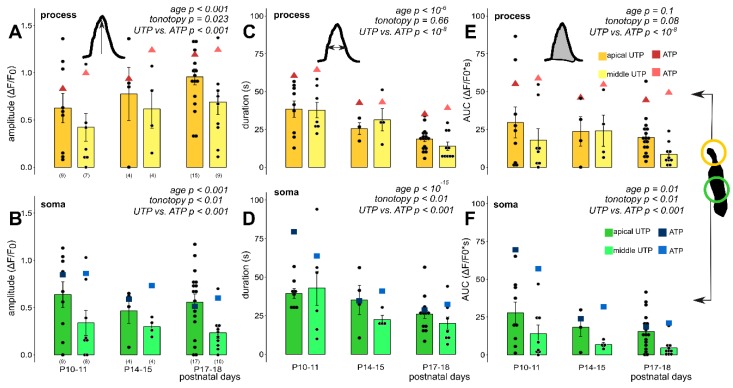
Exogenous UTP (100 µM in perfusion), a selective P2Y agonist, evoked developmental state- and tonotopy-dependent Ca^2+^ transients in Deiters’ cells in P10–18 mouse hemicochlea preparations. The bar graphs show the magnitudes of the amplitudes, duration and AUC values of all measured cell responses and their averages ± SEM in the processes (**A**,**C**,**E**) and somas (**B**,**D**,**F**) in the apical (darker colors) and middle (lighter colors) cochlear turns. Note the maturation-dependent gradual increase from P10–11 in the response amplitude in the processes (**A**) and its decline in the somas (**B**). The response durations and AUCs showed development-dependent gradual decrease in both the processes (**C**) and the somas (**D**). Significant tonotopic difference was measured between the amplitude of UTP responses in the phalangeal processes of Deiters’ cells in the apical vs. the middle cochlear turns (**A**) as well as in the somas (**B**). Turn difference in response duration was detected only in the somas (**D**), but not in the processes (**C**). The UTP induced responses were similar to ATP in their dependency on development and tonotopic location, but their amplitude and AUC were smaller and their duration was shorter. Averages of the respective values of ATP-evoked transients (from Figure 3 and Figure 4) are shown by the larger triangle and square symbols to assist easier comparison of UTP vs. ATP effects. Number of experiments are in parenthesis (**A**,**B**) and they are the same for the appropriate variables in C-F. Respective *p* values, showing the significance of age or tonotopy or the UTP vs. ATP difference are written in italics. Details of statistical analysis are given in the Materials and Methods.

Next, we applied the specific P2Y receptor agonist UTP onto the hemicochleae from the onset of hearing (P10) to the evolvement of the adult hearing to investigate the P2Y and P2X receptor contribution to the physiological purinergic signaling (P18; [4]). 100 µM UTP in the perfusion evoked Ca^2+^ transients in both subcellular compartments of the Deiters’ cells (Figure 5, Table 5) with similar age- and cochlear turn dependency, as ATP did (Figure 3C,D and Figure 4), which indicated that a mixed population of P2Y and P2X receptors are expressed throughout the Deiters’ cell plasma membrane. In the phalangeal processes, the amplitudes of the UTP responses gradually increased by developmental stages (*p* < 0.001; Figure 5A), while the transient amplitudes decreased gradually with age both in the apical and the middle turns in the soma of the Deiters’ cells (*p* < 0.001; Figure 5B). The tonotopic difference between the apical and the middle turns was significant both in the processes (*p* = 0.023) and the somas (*p* < 0.01). The response durations and AUC values gradually decreased by the development in both subcellular compartments in both cochlear turns (*p* < 10^−6^ for duration and *p* = 0.1 for AUC, process; *p* < 10^−15^ for duration and *p* < 0.01 for AUC, soma; Figure 5C–F), which suggests an increase in purinergic signaling efficiency and precision. There was no significant difference between UTP response durations and AUC values in the apical versus the middle cochlear turn in the phalangeal processes (*p* = 0.66 and *p* = 0.08, respectively; Figure 5C,E), similarly to the ATP response (Figure 4A,C), but a turn difference was observed in the case of the somas (*p* < 0.01 and *p* < 0.01; Figure 5D,F), unlike the ATP response (Figure 4B,D), which indicates that the receptor subtypes play an important role in tonotopic development.

We compared the absolute values of ATP- and UTP-evoked Ca^2+^ transients to assess the relative contribution of P2Y receptors to the total purinergic Ca^2+^ signaling. The UTP-induced Ca^2+^ responses had smaller amplitudes (*p* < 0.001, process; *p* < 0.001, soma) shorter response durations (*p* < 10^−8^, process; *p*< 0.001, soma) and smaller AUC values (*p* < 10^−8^, process; *p* < 0.001, soma; Figure 5, Table 5) than the ATP-evoked ones (Figure 3C,D, Figure 4 and Table 3 and Table 4). More importantly, the contribution of the P2Y receptor component was tonotopically diverse in both subcellular compartments because the majority of the purinergic response was P2Y-dependent in the apical turn (~75–100%; UTP_dF/F0_/ATP_dF/F0_ × 100), but only about 40–50% of the Ca^2+^ response was mediated by P2Y receptors in the middle turn in both the somas and the phalangeal processes (dF/F_0_ values taken from Table 3, Table 4, Table 5 and Table 6). The same finding, i.e., the dominance of P2Y receptors in the apical turn and about equal contribution of P2X and P2Y receptors in the middle turn is shown in the relation of the apical to the middle turn purinergic responses. The middle turn ATP transients were larger than (process; Figure 3C, Table 3) or similar to (soma; Figure 3D, Table 4) the apical turn ATP transients, while the UTP transients were larger in the apical turn in both subcellular compartments (Figure 5A,B, Table 5 and Table 6). As a tendency this was also true for the response durations and AUCs (Figure 4 vs. Figure 5C–F; Table 3 and Table 4 and Table 5 and Table 6).

## 4. Discussion

### 4.1. Postnatal Morphological Development of Deiters’ Cells in the Mouse Cochlea

The hearing organ of the mouse is immature at the time of birth and the newborn pups are deaf [40]. Several studies showed important structural and functional changes in the mouse cochlea from birth to the end of the third posnatal week when the adult-like hearing is attained [4,11].

The hemicochlea preparation preserves the anatomical features (e.g., membranes, spiral limbus, stria vascularis, organ of Corti) and allows for following the developmental processes in the classical cross sectional view in multiple turns of the cochlea. Morphological features of the organ of Corti have already been measured in the hemicochlea preparation [37,41,42,43]; however, the phalangeal processes of the Deiters’ cells are largely covered by the OHCs and the development of this subcellular compartment could not be explored. Biophysical studies and modelling supposed that the phalangeal processes have important roles in the cochlear micromechanics [32,44], thus we investigated the structural development of Deiters’ cells’ subcellular compartments in two different cochlear turns while using the hemicochlea preparation combined with single-cell electroporation dye-loading [30].

The soma of the Deiters’ cells reached the adult-like length around ~ P14 in mice [4] and we could not find tonotopic differencies. This is in accordance with the results in gerbils, where the length of Deiters’ cells in the apical, middle, and uppermiddle turns were very similar to each other. The length of the adult-like cells that we measured (45.96 ± 1.04 µm in the apical turn) was a bit longer than the one of the longest Deiters’ cells in the 129/SvEv mouse strain (33.7 ± 1.4 µm) [42]. However, cochlear dimensions differ between different mouse strains [42] and, according to our best knowledge, there is no morphological study addressing Deiters’ cells of the BALB/c mouse strain.

Elongation of the phalangeal process was slower and lasted longer. In all measured developmental stages, processes in the apical turn were longer, in correlation with the tonotopic rule. Phalangeal processes of the Deiters’ cells typically form tight junctions with the apical end of OHCs located one to three cells in the apical direction and in the adjacent lateral row [44,45]. This means that the length of the processes and nearby OHCs correlates. The length of the OHCs increases from the base to the apex by ~1.5 fold, which is a ~1.2 fold difference in the apical to middle turn relation [42]. At P20–25, our measurements from the Deiters’ cells’ phalangeal processes showed a bit higher difference (~1.5 fold) between the apical and middle turn. This could be a BALB/c strain specificity. Similarly to our findings, the OHCs were nearly as high in the adult as in the newborn gerbils, and only small and slow growth was detected that finished around the end of the third postnatal week. The space of Nuel between the OHCs and the processes of the Deiters’ cells appears around P6–10 (before the opening of the tunnel of Corti) in the place that was previously occupied by the immature, wider phalangeal processes [46,47,48]. We detected the narrowing of the processes that finished around P17–18.

### 4.2. Spontaneous Ca^2+^ Activity in Phalangeal Process and Soma of the Deiters’ Cells Decreases by Development with Different Pattern in the Middle and the Apical Cochlear Turns

Spontaneous Ca^2+^ activity and the spread of Ca^2+^ waves in the organ of Corti during the early stages of development are well-defined [7,9,49,50,51,52,53]. These Ca^2+^ spikes and waves are caused by ATP released from the supporting cells via connexin and/or pannexin hemichannels and its paracrine and autocrine action on purinergic receptors [54,55]. The Ca^2+^ spikes and waves have a major role in the maturation and pruning of hair cell synapses with the auditory nerves [9,25,53,56,57,58,59,60,61,62]. Most of these studies focused on the purinergic signaling-driven Ca^2+^ activity in the columnar shape supporting cells of the Kölliker’s organ present transiently in the GER during the first postnatal week. It is fundamental for IHC maturation and synapse formation, but the Ca^2+^ waves can also spread over the rows of the pillar cells to the LER, where Deiters’ cells are located [9,63,64,65]. The organotypic cochlear explant cultures that are primarily used in these studies allow for the visualization of cells and the Ca^2+^ waves spreading through them from the upper, scala media direction. The method is inadequate for the identification of Ca^2+^ signals in deeper cellular subregions. However, hemicochlea preparation with its classical cross section of the organ of Corti is suitable for subcellular imaging of spontaneous or extracellular ATP-evoked Ca^2+^ transients in Deiters’ cells. The use of the hemicochlea and the single cell electroporation loading of Ca^2+^ dye [30] allowed us to detect that the phalangeal processes have a higher tendency for spontaneous Ca^2+^ activity than the somata. The higher frequency of Ca^2+^ transients might be the consequence of the higher hemichannel density on the head plate of the phalangeal process [62]. That might cause a larger ATP release and concentration around the purinergic receptors on the head plate. It was shown that the endolymphatic surface of the cochlear cells express more purinergic receptors [29,66,67,68,69] and, in isolated cells (Hensen’s, young Deiters’ and hair cells), the intracellular Ca^2+^ waves started at the apical pole and then spread toward the basal part of the cell [29,68,70]. Although the intracellular Ca^2+^ waves might be the consequence of drug application [68], we can exclude this possibility in our experiments because the Ca^2+^ waves were spontaneously generated. The decline in the frequency of the spontaneous events in the Deiters’ cells, we measured, lasted longer (P20–25) than it was reported in the GER (till P10) [28]. The frequency of the spikes was lower than the ones that were observed in GER or LER [9]. The decreased spontaneous spike activity is probably caused by the age-dependent down regulation of purinergic receptors [27,28,66,71,72] or a shift in their sensitivity during their dynamic developmental expression [73]. A significant decrease in endogenous ATP release in parallel with the age-dependent down regulation of hemichannel proteins [61] cannot be excluded either.

Deiters’ cells in the apical cochlear turn showed an activity peak at P10–11. This is a critical developmental period for OHCs, which, this time start to express prestin, the motor protein of OHCs being in close connection with the Deiters’ cells [74,75]. The pruning and maturation of synapsis on OHCs are also intensive at this time [56,60]. Surprisingly, we did not detect this highly active period in Deiters’ cells in the middle turn. As the development of the cochlea starts from the basal part [76], we might miss the corresponding phase in the middle turn by not measuring at P8-9, only below (P5–7) and above (P10–11). A recent study at P0–13 demonstrated that OHCs also show spontaneous Ca^2+^ signals that are coordinated by the surrounding Deiters’ cells. They found the maximal OHC activity at P0 in both the basal and apical turns, which then gradually decreased and ultimately diminished at P5–6 [28].

### 4.3. Amplitude and Shape of Exogenous ATP-Induced Ca^2+^ Transients Depend on the Developmental Stage

The expression of purinergic receptors in the cochlea is strongly age-dependent. The amount of these receptors on Deiters’ cells elevates during the first postnatal week and then starts to decline after P12 [70,71,77,78,79,80,81,82]. This pattern in purinergic receptor expression might explain the measured changes in the amplitude, duration, and AUC of exogenous ATP-evoked transients in Deiters’ cell somas. First, these parameters increased from low levels at P5–7 to the highest ones at P10–11, followed by a subsequent gradual decrease in the P14–25 period. Turn differences were only recognized between transient amplitudes in the phalangeal processes, which may reflect the different distribution of purinergic receptors that are involved in the initiation of the Ca^2+^ response in this subcellular compartment. Contrary to the soma, the response amplitudes in the processes gradually increase by maturation. This might be the effect of the intracellular volume decrease on the dF/F_0_ values of the single wavelength indicator dye in the narrowing phalangeal processes. The uniform decrease in transient duration and AUC in all subcellular compartments and cochlear turns with maturation is probably the sign of the development of the Ca^2+^ buffering and removal mechanisms that make the shape of the transients sharper and less variable. The expression of Ca^2+^ buffers were only investigated in hair cells so far [83].

We should note that the basal turn was not included in our study (see Materials and Methods); therefore our results, although unequivocally show tonotopic differences, do not represent the whole tonotopic axis in the cochlea. Changes in Deiters’ cell morphology, spontaneous Ca^2+^ and purinergic responses we demonstrated in the context of subcellular location, maturation, and tonotopy cannot be extrapolated directly to the basal turn.

### 4.4. Both P2X and P2Y Receptors Are Involved in the Maturation-Dependent Purinergic Signaling, with Tonotopically Different Cellular Distribution

ATP activates both P2X and P2Y purinergic receptors [54], which are expressed on the Deiters’ cells [71]. We used UTP a P2Y receptor selective agonist in the critical P10–18 to test the P2Y-dependent effect w (onset of hearing–adult-like hearing) period.

The UTP-evoked Ca^2+^ responses were qualitatively similar to the ATP-evoked ones in age dependency of the amplitudes, duration, and AUC. However, the amplitude, response duration, and AUC values were smaller. This suggests that both P2X and P2Y receptors are present and functionally involved in the ATP evoked Ca^2+^ transients in the Deiters’ cells located in the apical and middle cochlear turns. Although the distribution of the P2X and P2Y receptors on the Deiters’ cells in the apical vs. the middle turn is probably different because the ratio of transient amplitudes measured in the two turns was rather the opposite, i.e., ATP evoked transients with slightly larger amplitude in the middle turn Deiters’ cells, while the UTP-induced responses were larger in the apical cells in all tested developmental periods.

## Figures and Tables

**Table 1:** Morphology of the developing Deiters’ cells. The length and width of the soma and the phalangeal process were measured in two turns of the cochlea (mean ± SEM).

	Soma				Process			
	Length (µm)		Width (µm)		Length (µm)		Width (µm)	
	Apical	Middle	Apical	Middle	Apical	Middle	Apical	Middle
P5–7	17.25 ± 0.97	27.58 ± 4.15	9.76 ± 0.89	8.15 ± 0.43	19.81 ± 0.84	13.54 ± 1.59	4.54 ± 0.44	3.95 ± 0.15
P10–11	42.25 ± 1.75	43.09 ± 0.69	9.42 ± 0.54	8.68 ± 0.43	15.08 ± 1.55	11.35 ± 0.55	3.61 ± 0.35	2.83 ± 0.16
P14–15	47.44 ± 1.23	46.23 ± 0.76	10.00 ± 0.51	8.03 ± 0.46	19.79 ± 0.67	14.9 ± 0.79	2.54 ± 0.24	1.85 ± 0.11
P17–18	44.88 ± 1.19	46.67 ± 1.17	9.91 ± 0.36	9.71 ± 0.37	20.14 ± 0.68	14.91 ± 0.64	1.55 ± 0.14	1.51 ± 0.06
P20–25	45.96 ± 1.04	44.51 ± 0.46	9.40 ± 0.36	8.95 ± 0.71	22.95 ± 1.36	15.28 ± 0.69	1.78 ± 0.14	1.14 ± 0.06

**Table 2:** Spontaneous intracellular Ca^2+^ activity in Deiters’ cell somas and phalangeal processes in the different ages and cochlear turns (10^−2^ events/min.; mean ± SEM).

	Soma		Process	
	Apical	Middle	Apical	Middle
P5–7	4.65 ± 2.26	8.80 ± 3.69	7.51 ± 4.34	9.04 ± 3.51
P10–11	47.14 ± 11.07	8.68 ± 4.96	56.92 ± 13.34	10.86 ± 6.12
P14–15	4.86 ± 1.56	4.10 ± 1.58	11.69 ± 3.60	6.10 ± 2.24
P17–18	1.59 ± 0.74	1.50 ± 1.04	3.94 ± 1.56	3.0 ± 1.55
P20–25	0 ± 0	6.70 ± 3.83	0.8 ± 0.6	12.11 ± 5.30

**Table 3:** Magnitude and kinetic characteristics of ATP (100 µM) induced Ca^2+^ transients in the phalangeal process of Deiters’ cells (mean ± SEM).

	Amplitude (dF/F_0_)		Duration (s)		AUC (s*dF/F_0_)	
	Apical	Middle	Apical	Middle	Apical	Middle
P5–7	0.92 ± 0.08	1.02 ± 0.17	41.41 ± 5.06	43.39 ± 5.26	44.85 ± 9.13	56.62 ± 12.56
P10–11	0.83 ± 0.12	0.99 ± 0.11	60.47 ± 8.13	64.45 ± 3.46	55.12 ± 12.26	58.80 ± 7.77
P14–15	0.93±0.09	1.23±0.08	42.57±2.90	43.01±3.61	46.04±5.29	54.64±5.59
P17–18	1.18±0.07	1.24±0.11	35.10 ± 2.63	39.13 ± 3.64	44.43 ± 4.92	49.3 ± 6.22
P20–25	1.28 ± 0.09	1.39 ± 0.13	29.15 ± 3.01	36.72 ± 4.52	42.41 ± 5.62	52.45 ± 8.17

**Table 4:** Magnitude and kinetic characteristics of ATP (100 µM) induced Ca^2+^ transients in the soma of Deiters’ cells (mean ± SEM).

	Amplitude (dF/F_0_)		Duration (s)		AUC (s*dF/F_0_)	
	Apical	Middle	Apical	Middle	Apical	Middle
P5–7	0.43 ± 0.10	0.56 ± 0.11	36.04 ± 3.88	46.82 ± 6.9	17.04 ± 4.37	31.05 ± 11.03
P10–11	0.84 ± 0.11	0.86 ± 0.10	79.48 ± 12.30	63.75 ± 3.76	69.46 ± 13.15	56.96 ± 8.84
P14–15	0.58 ± 0.05	0.73 ± 0.05	34.68 ± 2.79	40.90 ± 1.91	23.87 ± 3.33	31.74 ± 3.01
P17–18	0.51 ± 0.05	0.60 ± 0.06	28.82 ± 2.31	32.4 ± 3.14	18.39 ± 2.53	21.10 ± 3.10
P20–25	0.56 ± 0.07	0.48 ± 0.10	27.94 ± 3.16	25.17 ± 2.57	19.95 ± 4.49	14.18 ± 4.05

**Table 5:** Magnitude and kinetic characteristic of UTP (100 µM) induced Ca^2+^ transients in the phalangeal process of Deiters’ cells (mean ± SEM).

	Amplitude (dF/F_0_)		Duration (s)		AUC (s*dF/F_0_)	
	Apical	Middle	Apical	Middle	Apical	Middle
P10–11	0.62 ± 0.15	0.42 ± 0.14	38.41 ± 5.44	37.73 ± 5.51	29.57 ± 10.21	17.89 ± 7.50
P14–15	0.77 ± 0.28	0.61 ± 0.20	25.51 ± 4.42	31.39 ± 7.45	23.67 ± 9.47	24.07 ± 10.27
P17–18	0.95 ± 0.08	0.68 ± 0.13	18.52 ± 1.78	13.9 ± 2.78	12.37 ± 3.00	8.50 ± 2.27

**Table 6:** Magnitude and kinetic characteristic of UTP (100 µM) induced Ca^2+^ transients in the soma of Deiters’ cells (mean ± SEM).

	Amplitude (dF/F_0_)		Duration (s)		AUC (s*dF/F_0_)	
	Apical	Middle	Apical	Middle	Apical	Middle
P10–11	0.63 ± 0.13	0.33 ± 0.13	39.41 ± 3.34	42.9 ± 12.79	27.81 ± 7.20	13.93 ± 8.86
P14–15	0.46 ± 0.13	0.29 ± 0.09	35.13 ± 9.53	22.54 ± 2.63	18.27 ± 6.06	6.80 ± 1.32
P17–18	0.55 ± 0.08	0.23 ± 0.06	25.99 ± 3.1	20.06 ± 5.06	15.47 ± 3.01	4.51 ± 1.77
